# Sutureless FOCUS harmonic scalpel versus clamp-and-tie techniques for thyroidectomy: a meta-analysis of 43 randomized controlled trials

**DOI:** 10.1097/JS9.0000000000002113

**Published:** 2024-10-07

**Authors:** Roberto Cirocchi, Matteo Matteucci, Eleonora Lori, Vito D’Andrea, Alberto Arezzo, Daniele Pironi, Stefano Avenia, Justus Randolph, Ileana Tiraboschi, Giovanni Domenico Tebala, Georgi I. Popivanov, Salvatore Sorrenti

**Affiliations:** a Department of General Surgery, University of Perugia, Terni; b Department of General Surgery, University of Milan, Milan; c Department of Surgery, Sapienza University of Rome, Rome; d Department of Surgical Sciences, University of Torino, Turin, Italy; e Georgia Baptist College of Nursing, Mercer University, Atlanta, GA, USA; f Division of General and Hepatobiliary Surgery, Department of Surgical Science, Dentistry, Gynecology and Pediatrics, University of Verona, Verona; g Department of Digestive and Emergency, Hospital of Terni, Terni, Italy; h Department of Surgery, Medical Military Academy of Sofia, Sofia, Bulgaria

**Keywords:** clamp-and-tie technique, EBDs, FOCUS harmonic scalpel, thyroidectomy

## Abstract

**Background:**

One of the most important surgical steps during thyroidectomy is the safe ligation of vessels. In fact, it is crucial to avoid postoperative bleeding and nerves’ injury. The “clamp and tie” technique was first introduced in the 19^th^ century. Since then, a lot of other techniques have been adopted to facilitate the safe ligation and sectioning of thyroidal vessels; however, one of the most relevant advances is the introduction of energy-based devices (EBDs), which occurred three decades ago.

**Purpose:**

The aim of this systematic review and meta-analysis is to evaluate the safety and effectiveness of sutureless FOCUS harmonic scalpel (second-generation EBDs) versus conventional clamps-and-tie technique) in total thyroidectomy.

**Results:**

This systematic review and meta-analysis represent the largest comparison in the literature between the thyroidectomy procedure with the second-generation advanced harmonic scalpel ultrasonic focus (UAS FOCUS) versus the conventional clamp-and-tie (CT) technique: as a matter of fact, it includes 43 randomized controlled trials (RCTs) and a total of 10 361 patients. The incidence of transient recurrent laryngeal nerve palsy was statistically lower in patients undergoing UAS (3.99%) rather than CT (5.23%) (RR 0.79, 95% CI 0.63–0.99). The incidence of transient hypocalcemia was 11.3% in patients who underwent thyroidectomy with UAS FOCUS and 15.4% in those who underwent thyroidectomy with CT.

**Conclusion:**

Sutureless EBD is associated with a lower risk of damage to the laryngeal nerves and parathyroid glands. The rate of hypocalcemia and nerve paresis is indeed lower due to less thermic damage. Sutureless EBD can also diminish the rate of postoperative bleeding and cervical hematoma, a rare but potentially fatal complication.

## Introduction

Highlights

- Sutureless FOCUS harmonic scalpel versus clamp-and-tie techniques for thyroidectomy.
- Sutureless EBD is associated with a lower risk of damage to the laryngeal nerves, parathyroid glands, postoperative bleeding and neck hematoma.

The rate of thyroidectomies has increased significantly in recent years, largely due to an increase in the number of diagnostic procedures performed to detect potential small thyroid cancers^1^. In the United States, the rate of thyroidectomies exhibits a 6.2-fold variation compared to that of prostatectomy, which is generally regarded as a surgical procedure with a high degree of variability^2^.

The thyroid gland is highly vascularized and the presence of variations in its vascular supply are often found. Moreover, the proximity of the vessels to the laryngeal nerves increases the risk of laryngeal nerve injury. Therefore, vascular safe ligation is of critical importance to avoid postoperative bleeding and nerve injury, which can lead to considerable morbidity and transient or permanent disability.

Thyroidectomy is the most effective therapeutic option for the treatment of thyroid cancer, goiter and hyperthyroidism. The technique of thyroidectomy was first standardized by Albert Theodor Billroth (1829–1894) and Emil Theodor Kocher (1841–1917)^3^ who proposed performing the procedure in two stages. Initially the thyroid arteries on each side were ligated, then the gland was excised. This surgical technique is usually referred to as clamp-and-tie (or cut-and-tie)^4^. Sutureless energy-based devices (EBDs) have been introduced in contemporary clinical practice to facilitate the rapid and safe ligation and sectioning of thyroidal vessels. The sutureless EBD combines both pressure and energy to the vascular wall in order to coagulate the medium-sized vessels prior to their sectioning. The use of sutureless EBDs during thyroidectomy can result in a reduction in intraoperative time and in the prevention of the transmission of thermal energy to nearby tissues, thereby reducing the risk of thermal-induced damage to the nerves and the surrounding tissues. In addition, shorter operative times can lead to improved patient outcomes, faster recovery times, and prove to be cost-saving for both the operative procedure and the hospital overall^5,6^.

Over the past two decades, numerous randomized controlled trials (RCTs) and controlled clinical trials (CCTs) have been conducted on the use of sutureless devices during thyroidectomy. However, the collective findings of systematic reviews and meta-analyses have been found to be highly heterogeneous^7–17^. We hypothesize that the discrepancy observed in previous reviews may be attributed to the inclusion of numerous low-quality studies with a high degree of bias. Consequently, this systematic review and meta-analysis will address the potential shortcomings of previous meta-analytic research by including only the results of RCTs. In summary, this systematic review and meta-analysis will assess the safety and efficacy of the second-generation sutureless EBDs (FOCUS harmonic scalpel) in comparison to the conventional clamp-and-tie technique during thyroidectomy, as evidenced in RCTs.

## Methods

### Criteria for considering studies for this systematic review and meta-analysis

Types of studies: RCTs were included in our analysis.

Types of participants: We only considered studies involving adult patients, aged 18 and older, who had undergone either partial or total thyroidectomy.

Types of interventions: Thyroidectomy with second-generation UAS Focus (“Harmonic scalpel”, Johnson & Johnson) compared to clamp-and-tie (CT) thyroidectomy, which includes procedures performed using the traditional ligature technique or with the use of bipolar forceps or electrosurgery.

Summary of specific exclusion criteria: We have excluded studies whose participants underwent extended tracheal resection, extended esophageal resection and extended neck vascular resection.

Primary outcomes: Permanent laryngeal superior and recurrent inferior laryngeal nerve palsy, permanent hypocalcemia and the need for re-intervention due to bleeding.

Secondary outcomes: Transient laryngeal superior nerve palsy, transient recurrent inferior laryngeal nerve palsy, transient hypocalcemia, surgical infection of the wound, neck hematoma, neck seroma, operative time and length of postoperative hospitalization.

Method of outcome measurement: The nerve is used as the unit of analysis for nerve-related outcomes, such as transient and permanent nerve palsy, whereas the participant is the unit of analysis for all other outcomes.

### Search methods for identification of studies

In order to identify all relevant sources, exhaustive research was carried out through many databases without time limits. We did not place any restrictions on the language of publication. The following databases were searched: MEDLINE, SCOPUS, Web of Science, and ClinicalTrials.gov (SDC1). Furthermore, we endeavored to identify any additional studies or ancillary publications that might meet the eligibility criteria. In addition, the lists of bibliographic references of the included studies were thoroughly reviewed; likewise, the reference lists of systematic reviews, meta-analyses and health technology assessment reports were checked. The study was conducted in accordance with the PRISMA (Preferred Reporting Items for Systematic Reviews and Meta-Analyses) and AMSTAR (Assessing the Methodological Quality of Systematic Reviews) guidelines^18,19^.

### Data collection and analysis

Two reviewers (R.C., V.D.A.) separately assessed the abstracts and titles of each record from the literature searches to select studies for additional evaluation. The full texts of all potentially relevant records were then acquired. For studies that fulfilled our inclusion criteria, two reviewers (R.C., M.M.) independently extracted essential participant and intervention details. We outlined the interventions following the ‘Template for Intervention Description and Replication’ (TIDieR) checklist^20,21^.

### Assessment of risk of bias in included studies

For each included study, two reviewers (M.M., R.C.) independently assessed the risk of bias. Disagreements were resolved by consensus; if consensus could not be reached, a third reviewer (V.D.A.) was consulted. The review team then reached a decision by consensus. We used the Cochrane Risk of Bias tool (Higgins 2019b), useful for classifying the risk of bias into three categories (low, high or unclear). The criteria and classifications outlined in the Cochrane Handbook for Systematic Reviews of Interventions were used to assess each bias item^22^.

### Measures of treatment effect

When at least two included trials were available to compare a specific outcome, we reported dichotomous data as risk ratios (RR) with 95% CI. We estimated the intervention effect using mean differences (MD) with 95% CIs for continuous outcomes measured on the same scale. When there was considerable clinical or methodological heterogeneity, we refrained from presenting trial results as a pooled effect estimate in a meta-analysis. Heterogeneity (inconsistency) was assessed by visual inspection of forest plots and a chi-square test with a significance level of 75%^23–25^.

## Results

**Figure 1:**
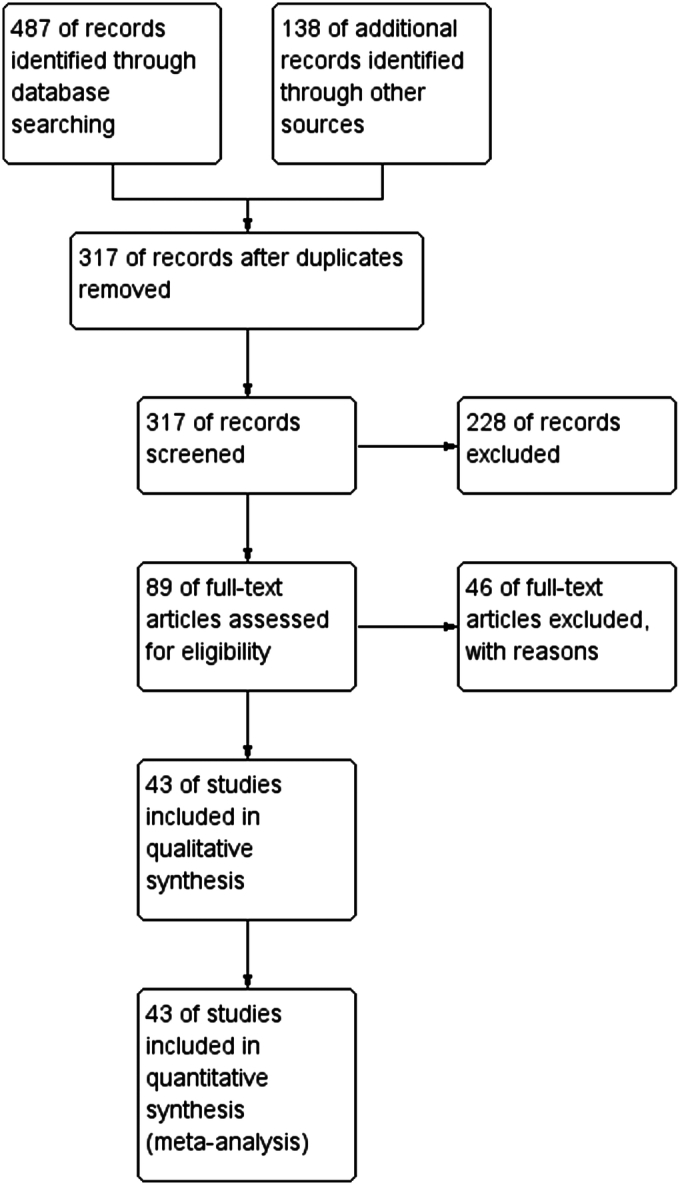
Studies included in qualitative and quantitative analysis.

The initial literature search yielded 645 articles. After the removal of duplicates and a preliminary screening, the full text of 89 articles was reviewed to determine their eligibility for inclusion in the meta-analysis. In total, 43 RCTs comparing thyroidectomy with the second-generation Johnson & Johnson UAS Focus (“Harmonic Scalpel”) to the conventional CT technique were included. (Fig. 1).

The following are the main features of the included studies:

The first table (Table 1) reports the authors, their nationality, year of publication of the study, number of study centers, duration and number of patients enrolled, divided by type of intervention, i.e. whether they underwent thyroidectomy with UAS Focus or with CT thyroidectomy.
Participants’ characteristics such as age, sex, BMI, and diagnosis are reported in Table 2. The characteristics are always divided between UAS and CT.
In the third table (Table 3), the studies reviewed are classified according to the type of intervention: total thyroidectomy, partial thyroidectomy, lobectomy and whether cervical lymph node dissection was also performed (unilateral and bilateral).

**Table 1:** RCTs included in systematic review and meta-analysis.

References	Nation	No. centers	Time of enrollment	No. patients included		Grant or other financial support
				HS	CT	
Memon *et al.* ^26^	Pakistan	1	2020	35	35	No
Basha^27^	Egypt	1	2020–2021	30	30	NR
Haq *et al.* ^28^	India	2	2018–2020	50	50	No
Soliman M. *et al.* ^29^	Egypt	1	2019–2020	38	38	No
Amer *et al.* ^30^	Egypt	1	2016–2019	50	42	No
El Sherpiny^31^	Egypt	1	2018–2019	30	30	No
Elghany *et al.* ^32^	Egypt	1	2016–2019	100	100	No
Saim *et al.* ^33^	Pakistan	1	2018–2019	31	31	NR
Buzdar *et al.* ^34^	Pakistan	1	2019–2020	47	47	NR
Bajaj *et al.* ^35^	India	1	2017–2018	30	30	No
Ali *et al.* ^36^	Pakistan	1	2016–2017	30	30	NR
Noori *et al.* ^37^	Iraq	1	2014–2017	32	32	No
Maeda *et al.* ^38^	Japan	1	2013–2016	21	24	Yuasa Memorial Foundation
Ahmed *et al.* ^39^	Pakistan	1	2016–2017	30	30	No
Anandaravi *et al.* ^40^	India	1	2016	19	15	No
Zehra *et al.* ^41^	Pakistan	1	2015–2016	38	38	NR
Saloom *et al.* ^42^	Iraq	3 Baghdad teaching hospital, Al-Hayat hospital and Al-Mukhtar hospital)	2011–2016	105	104	NR
Su *et al.* ^43^	China	1	2013–2016	167	180	NR
Gaber *et al.* ^44^	Egypt	1	2015	20	20	NR
Arslan *et al.* ^45^	Turkey	2	2013–2015	101	105	No
Blanchard *et al.* ^46^	France	13	2012–2014	670	659	French Ministry of Health
Shaaban *et al.*2017^47^	Egypt	1	NR	25	25	No
Basurto-Kuba *et al.* ^48^	Mexico	1	NR	50	50	NR
Al-Dhahiry *et al.* ^49^	Iraq	1	2012–2015	26	26	No
Minni *et al.* ^50^	Italy	3	2008–2014	174	187	NR
Aziz *et al.* ^51^	Pakistan	2	2014	80	80	No
Karaca *et al.* ^52^	Turkey	2	2007–2012	468	461	NR
Cannizzaro *et al.* ^53^	Italy	1	2011–2013	141	124	NR
Yener *et al.* ^54^	Turkey	1	2011	42	43	NR
Ciftci^55^	Turkey	1	2011	38	38	NR
Zanghì *et al.* ^56^	Italy	1	2010–2011	41	42	No
Bangash *et al.* ^57^	Pakistan	1	2010–2012	60	60	NR
Duan *et al.* ^58^	China	1	2009–2012	389	389	NR
Soroush *et al.* ^59^	Iran	1	NR	33	35	Tehran University of Medical Sciences grant
Konturek *et al.* ^5^	Poland	1	2010–2011	41	41	NR
Sista *et al.* ^60^	Italy	1	2008–2011	130	131	NR
Ferri *et al.* ^61^	Italy	2	2010–2011	50	50	NR
Materazzi *et al.* ^62^	Italy	2 (Pisa, Grosseto)	2006–2011	141	127	NR
Gentileschi *et al.* ^63^	Italy	1	2010	43	38	NR
He *et al.* ^64^	China	1	2010	51	54	NR
Mourad *et al.* ^65^	Belgium, Italy	4	2008	34	34	NR
Askar *et al.* ^66^	Egypt	1	2008–2010	65	65	NR
Di Renzo *et al.* ^67^	Italy	1	2009	31	31	No

CT, clamp-and-tie; NR, not reported; HS, harmonic scalpel; RCT, randomized controlled trial.

**Table 2:** Characteristics of patients enrolled in the RCTs.

References	Age mean (SD)		Sex (M/F)		BMI mean (SD)		Cancer		Goiter (multinodular, toxic)		Other pathologies	
	HS	CT	HS	CT	HS	CT	HS	CT	HS	CT		
Memon *et al.* ^26^	41.9±8.8		M39/F31		NR	NR	NR	NR	NR	NR	NR	
Basha^27^	35.4±5.2	36.2±4.8	M4/F26	M3/F27	26.4±2.2	26.7±2.1	14	12	17	14	Adenoma/inflammatory disease	
											HS 6	CT 6
Haq *et al.* ^28^	39.8	43.8	M20/F30	M23/F27	NR	NR	NR	NR	NR	NR	NR	
Soliman M. *et al.* ^29^	39±11.3 (21–57)	39.6±10.8 (25–55)	M6/F32	M4/F34	NR	NR	0	0	0	0	Simple nodular goiter and/or solitary thyroid nodule	
											HS 38	CT 38
Amer *et al.* ^30^	42.53±12.139	45.74±13.95	M4/F46	M5/F37	NR	NR	0	0	50	42	0	
El Sherpiny^31^	44.7	46.8	M5/F25	M10/F20	NR	NR	0	0	30	30	0	
Elghany *et al.* ^32^	40±10	39±9	M22/F78	M16/F84	NR	NR	NR	NR	NR	NR	NR	
Saim *et al.* ^33^	45.29±4.56	44.96±2.97	M64.5%/F35.5%	M74.2%/F25.8%	NR	NR	0	0	31	31	0	
Buzdar *et al.* ^34^	37.26±5.50 (23–50)	37.26±5.50 (23–50)	M21/F26	M24/F23	NR	NR	NR	NR	NR	NR	NR	
Bajaj *et al.* ^35^	47.7 (20–72)	52.4 (22–71)	M8/F12	M7/F13	NR	NR	4	3	24	25	NR	
											HS 2	CT 2
Ali *et al.* ^36^	31.50±8.89	36.20±10.98	M5/F25	M4/F26	NR	NR	0	0	Benign thyroid disease, including: nodular, multinodular goiter, thyroid cyst, toxic adenoma			
									HS 30		CT 30	
Zehra *et al.* ^41^	31.92±6.48	32.71±5.78	13 (34.21) (M) 25 (65.79) (F)	12 (31.58) (M) 26 (68.42) (F)	NR	NR	0	0	38	38	0	
Noori *et al.* ^37^	45.7	47.8	M4/F28	M6/F26	NR	NR	3	5	26	23	Hashimoto’s thyroiditis	
											HS 3	CT 4
Maeda *et al.* ^38^	51.5±11.84	57.8±13.73	M4/F17	M5/F19	23.6±3.42	23.7±3.93	14	16	7	8	0	
Ahmed *et al.* ^39^	34.73±10.22	30.93±12.29	M9/F21	M13/F17	NR	NR	0	0	30	30	NR	
Gaber *et al.* ^44^	38.00±8.17 Range 23–60	35.15±6.81 Range 25–50	5 (25.0) (M) 15 (75.0) (F)	7 (35.0) (M) 13 (65.0) (F)	NR	NR	0	0	20	20	0	
Su *et al.* ^43^	43.2 ± 4.1 years.	43.2 ± 4.1 years.	41 M–486 F	41 M–486 F	NR	NR	298	298	98	98	131 thyroid adenoma	
Anandaravi *et al.* ^40^	30/50	30/50	M5/F29		NR	NR	1		33		0	
Arslan *et al.* ^45^	43	47	M20/F81	M16/F89	NR	NR	25	20	76	85	0	
Blanchard *et al.* ^46^	51.1 (40.9–61.9)	51.3 (40.0–61.2)	M137/F533	M130/F529	25.5 (22.8–29.4)	25.9 (22.4–29.4)	150	141	520	518	0	
Shaaban et al.^47^	39.6±10.8 (21–57)	39±11.3 (25–55)	M3/F22	M4/F21	NR	NR	0	0	25	25	0	
Basurto-Kuba *et al.* ^48^	NR	NR	NR	NR	NR	NR	NR	NR	NR	NR	NR	
Saloom et al.^42^	49±12	48±14	12 (11.4%) (M) 93 (88.6%) (F)	14 (13.5%) (M) 90 (86.5%) (F)	NR	NR	6 (5.7%)	5 (4.8%)	12 (11.4%)	9 (8.7%)	NON TOXIC MNG HS 87 (82.9%	NON TOXIC MNG CT 90 (86.5%)
Al-Dhahiry *et al.* ^49^	41	35	M6/F20	M6/F20	NR	NR	1	1	25	25	0	
Minni *et al.* ^50^	>18	>18	M73/F101	M79/F108	NR	NR	0	0	174	187	0	
Aziz *et al.* ^51^	42±9	43±9,8	M22/F134		NR	NR	0	0	80	80	0	
Karaca *et al.* ^52^	47.61±13.68	48.41±12.98	NR	NR	NR	NR	32		897		NR	
Ciftci^55^	49±14	46±13	12 (31.5) (M) 26 (68.5) (F)	12 (31.5) (M) 26 (68.5) (F)	NR	NR	8 (21.0)		8 (21.0)		29 (76.3)	
Cannizzaro *et al.* ^53^	53	53	M/F ratio=1:6,25		NR	NR	0	0	141	124	0	
Yener *et al.* ^54^	43.6	37.4	M6/F36	M7/F36	NR	NR	0	0	42	43	0	
Zanghì *et al.* ^56^	NR	NR	NR	NR	NR	NR	0	0	41	42	0	
Bangash *et al.* ^57^	38.6 (±4.3)	35.3 (±2.6)	11:1 M:F	9:1 M:F	NR	NR	1	0	33 (55%)	41 (68.33%)	Colloid goiter (%) 14 (23.3) 11 (18.3) Diffuse goiter (%) 13 (21.6) 8 (13.3)	
Duan *et al.* ^58^	50.1	48.5	M66/F323	M57/F332	NR	NR	56	64	324	318	Hashimoto’s thyroiditis	
											HS 9	CT 7
Materazzi *et al.* ^62^	51.68±12.2	53.97±12.5	112 (F), 29 (M)	92 (F), 35 (M)	NR	NR	NR	NR	141	127	0	
Soroush *et al.* ^59^	38.7±13.5	43.2±14.5	M16/F17	M19/F16	NR	NR	9	16	22	17	NR	
											HS 2	CT 2
Konturek *et al.* ^5^	41.1	42	M7/F34	M8/F33	NR	NR	0	0	41	41	0	
Sista *et al.* ^60^	49.3	51.1	M31/F99	M29/F102	NR	NR	24	24	99	98	Thyroiditis	
											HS 7	CT 8
Ferri *et al.* ^61^	48.7	51.4	M22/F28	M19/F31	NR	NR	6	4	44	46	0	
Gentileschi *et al.* ^63^	49	48	M9/F34	M4/F34	NR	NR	28	27	15	11	0	
He *et al.* ^64^	48	46.5	M6/F45	M7/F47	22.8	22.6	51	54	0	0	0	
Mourad *et al.* ^65^	50	47	M8/F26	M8/F26	NR	NR	0	0	34	34	0	
Askar *et al.* ^66^	41.81±13.4 (16–79)	36.24 ±12.62 (12–72)	M12/F54	M16/F49	NR	NR	0	0	47	46	Thyrotoxicosis	
											HS 18	CT 19
Di Renzo *et al.* ^67^	50.5±12.1	51.5±13.7	M8/F23	M7/F24	NR	NR	0	0	31	31	0	

CT, clamp-and-tie; F, female; HS, harmonic scalpel; M, male; NR, not reported; RCT, randomized controlled trial.

**Table 3:** Type of intervention performed in the enrolled patients.

References	Total thyroidectomy		Subtotal thyroidectomy		Lobectomy		Lateral lymphadenectomy	
	HS	CT	HS	CT	HS	CT	HS	CT
Memon *et al.* ^26^	35	35	0	0	0	0	0	0
Basha^27^	30	30	0	0	0	0	NR	NR
Haq *et al.* ^28^	50	50	0	0	0	0	0	0
Soliman M. *et al.* ^29^	38	0	0	0	0	0	0	0
Amer *et al.* ^30^	50	42	0	0	0	0	0	0
El Sherpiny^31^	30	30	0	0	0	0	0	0
Elghany *et al.* ^32^	100	100	0	0	0	0	0	0
Saim *et al.* ^33^	31	31	0	0	0	0	0	0
Buzdar *et al.* ^34^	47	47	0	0	0	0	NR	NR
Bajaj *et al.* ^35^	30	30	0	0	0	0	0	0
Al.i *et al.* ^36^	0	2	19	9	11	19	0	0
Noori *et al.* ^37^	NR	NR	NR	NR	NR	NR	NR	NR
Maeda *et al.* ^38^	6	10	0	0	15	14	NR	NR
Zehra *et al.* ^41^	38	38	0	0	0	0	0	0
Ahmed *et al.* ^39^	NR	NR	NR	NR	NR	NR	0	0
Anandaravi *et al.* ^40^	1		33		0		1	
Arslan *et al.* ^45^	101	105	0	0	0	0	0	0
Blanchard *et al.* ^46^	670	659	0	0	0	0	11	18
Shaaban *et al.* ^47^	25	25	0	0	0	0	0	0
Basurto-Kuba *et al.* ^48^	NR	NR	NR	NR	NR	NR	NR	NR
Su *et al.* ^43^	47	60	0	0	45	31	21 54 + unilateral central lymph node dissection	23 66 + unilateral central lymph node dissection
Gaber *et al.* ^44^	20	20	0	0	0	0	0	0
Al.-Dhahiry *et al.* ^49^	26	26	0	0	0	0	0	0
Minni *et al.* ^50^	174	187	0	0	0	0	0	0
Aziz *et al.* ^51^	80	80	0	0	0	0	0	0
Sal.lom *et al.* ^42^	105	104	0	0	0	0	0	0
Karaca *et al.* ^52^	468	461	0	0	0	0	0	0
Ciftci^55^	38	38	0	0	0	0	0	0
Cannizzaro *et al.* ^53^	141	124	0	0	0	0	0	0
Yener *et al.* ^54^	22	26	0	0	21	16	NR	NR
Zanghì *et al.* ^56^	41	42	0	0	0	0	0	0
Bangash *et al.* ^57^	60	60	0	0	0	0	0	0
Duan *et al.* ^58^	389	389	0	0	0	0	0	0
Soroush *et al.* ^59^	33	35	0	0	0	0	NR	NR
Materazzi *et al.* ^62^	141	127	0	0	0	0	0	0
Konturek *et al.* ^5^	41	41	0	0	0	0	0	0
Sista *et al.* ^60^	119	122	0	0	11	9	0	0
Ferri *et al.* ^61^	50	50	0	0	0	0	0	0
Gentileschi *et al.* ^63^	43	38	0	0	0	0	0	0
He *et al.* ^64^	51	54	0	0	0	0	51	54
Mourad *et al.* ^65^	34	34	0	0	0	0	0	0
Askar *et al.* ^66^	65	65	0	0	0	0	0	0
Di Renzo *et al.* ^67^	31	31	0	0	0	0	0	0

CT, clamp-and-tie; HS, harmonic scalpel; NR, not reported; RCT, randomized controlled trial.

The analysis shows that the total number of participants in the included studies was 10.361: 5.152 patients underwent thyroidectomy with UAS FOCUS (49%) and 5.209 were treated with conventional CT technique (51%). The main type of surgery was total thyroidectomy, which was performed in 9.097 patients (87%); subtotal thyroidectomy was performed in 135 participants (1.3%) and lobectomy was performed in 298 patients (2.8%). The age of the patients ranged from 18 to 79 years and most of them were female (72%).

### Quality assessment of the included studies

**Figure 2:**
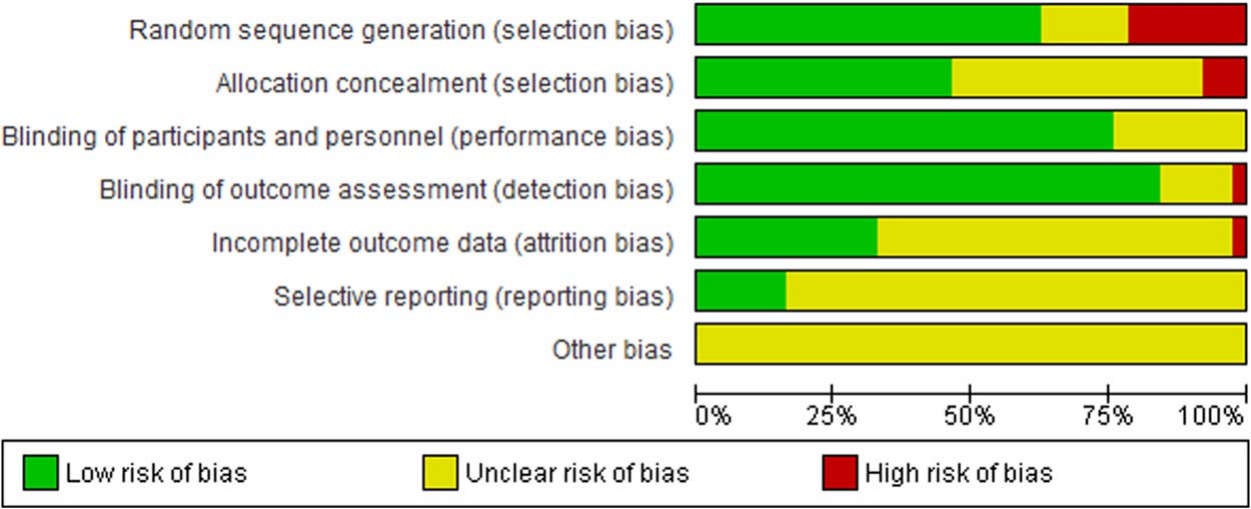
Risk of bias graph: review authors’ judgements about each risk of bias item presented as percentages across all included studies.

**Figure 3:**
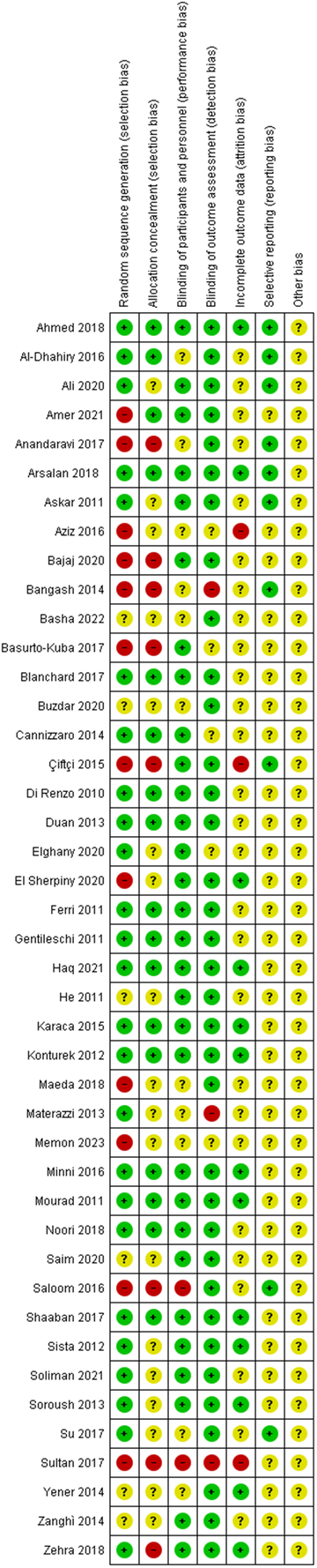
Risk of bias summary: review authors’ judgements about each risk of bias item for each included study.

The risk of bias graph and summary are shown in Figure 2 and Figure 3, respectively.

In the risk of bias assessment, random sequence generation was the first considered item: 27 of the included studies showed a low risk of bias in this domain; 8 of the studies showed a high risk of bias and 8 studies had an unclear risk due to the missing information on random sequence generation.

In the allocation concealment: 20 of the included studies showed a low risk of bias in this area; as a matter of fact, in most cases the assignments were concealed in opaque envelopes, that were sealed and inserted into patient files. In 3 studies there was a high risk of bias, while 20 studies showed an unclear risk due to a lack of information on allocation concealment.

In the blinding of participants and personnel: 33 of the studies had a low risk of bias, while 10 studies an unclear risk.

In blinding of outcome assessment: 37 of the included studies had a low risk of bias, only 1 study showed a high risk, and 5 studies an unclear risk due to lack of information in this area.

In incomplete outcome data, only one study presented a high risk of bias in this item; 17 studies showed a low risk, while 25 studies had an unclear risk due to missing of information on patients lost to follow-up.

In selective reporting none of the studies showed a high risk: 12 studies had a low risk of bias, while 31 showed an unclear risk.

### Primary outcomes

#### Permanent inferior laryngeal nerve palsy

Permanent recurrent inferior laryngeal nerve palsy was defined as an injury detected either clinically, by laryngoscopy or both at 6 months or later after thyroidectomy. The incidence of permanent laryngeal nerve palsy was assessed in 31 RCTs, analyzing 6524 patients (3288 with UAS and 3236 with CT); permanent laryngeal nerve palsy occurred in 0.57% of patients who underwent thyroidectomy with UAS (19/3.288) compared to 0.67% of patients who underwent thyroidectomy with CT (22/3.236). Considering these results, we can say that no advantage has been reported in patients undergoing thyroidectomy with UAS compared to the conventional CT technique (RR 1.11, 95% CI 0.51–2.42).

#### Permanent hypocalcemia

Permanent hypocalcemia was defined as the permanent need of supplement therapy (calcium and/or vitamin D) after at least 6 months after thyroidectomy or a total serum calcium concentration lower than a cut-off of 8 mg/dl. The incidence of permanent hypocalcemia was evaluated in 32 RCTs, analyzing 6689 patients (3364 UAS and 3325 CT); permanent hypocalcemia was 0.92% in patients who underwent thyroidectomy with UAS (31/3364) compared to 1.26% in patients who underwent thyroidectomy with CT (42/3325). In the light of these results, we can say that there is no statistically significant advantage in patients who underwent thyroidectomy with UAS compared to those who underwent the conventional CT technique (RR 0.80, 95% CI 0.50–1.28).

#### Permanent superior laryngeal nerve palsy

Permanent superior laryngeal nerve palsy was defined as a clinical diagnosis of injury detected by clinical evaluation, laryngoscopy or both, by assessing vocal cord movement at six months after surgery. This outcome was analyzed in 3 RCTs considering 672 patients (326 UAS and 346 CT); there were a total of 5 cases of permanent palsy of the superior laryngeal nerve (3 UAS and 2 CT). For the UAS technique, the cases were 0.9% (3/326) while for the CT technique the cases were 0.5% (2/346). Also, in this case we found no statistically decisive differences between the two methods (RR 1.56, 95% CI 0.27–9.14).

#### Postoperative bleeding requiring re-intervention

Cervical re-exploration may be necessary for active bleeding or for neck hematoma. Postoperative bleeding was reported in 9 RCTs for a total of 1912 participants (964 UAS and 948 CT). The risk of re-operative bleeding was 0.21% for UAS (2/964) compared with 0.73% for CT (7/948). No statistically significant advantage was reported in patients operated with UAS technique compared to CT technique (RR 0.47, 95% CI 0.14–1.58).

### Secondary outcomes

#### Transient inferior laryngeal nerve palsy

The incidence of transient laryngeal nerve palsy was evaluated in 34 RCTs, which analyzed 5959 patients (2981 with UAS and 2978 with CT); in patients who underwent thyroidectomy with UAS (119/2981) transient laryngeal nerve palsy was 3.99% compared to 5.30% in patients who underwent thyroidectomy with CT (158/2978). From the data collected, it is possible to observe that a statistically significant advantage has been reported in patients who underwent thyroidectomy with UAS compared to patients who underwent thyroidectomy with CT (RR 0.79, 95% CI 0.63–0.99).

#### Transient hypocalcemia

The incidence of transient hypocalcemia was reported in 36 RCTs, which reported 7041 patients (3536 with UAS and 3505 with CT); transient hypocalcemia was 11.3% in patients undergoing thyroidectomy with UAS (403/3536) compared to 15.4% in patients undergoing CT thyroidectomy (542/3505). The data collected showed that patients who underwent thyroidectomy with UAS had a statistically significant benefit compared to those who underwent conventional CT (RR 0.67, 95% CI 0.56–0.81).

#### Transient superior laryngeal nerve palsy

Temporary superior laryngeal nerve palsy was analyzed in 5 RCTs considering 1083 patients (525 UAS and 558 CT); the cases of temporary paralysis were 1.9% (10/525) for UAS compared to 2.1% (12/558) for CT. Considering the data obtained, it is clear that there is no significant difference between the two techniques (RR 0.95, 95% CI 0.32–2.77).

#### Neck hematoma

**Figure 4:**
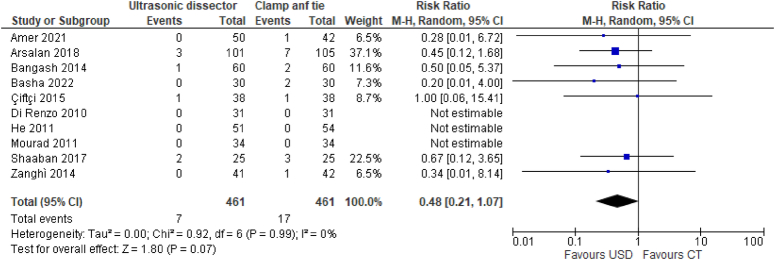
Hematoma of neck.

The presence of cervical hematoma was reported in 10 RCTs for a total of 922 participants (461 UAS and 461 CT), the prevalence of hematoma was 1.5% for the UAS technique (7/461) versus 3.6% for the CT technique (17/461). Thyroidectomy with UAS technique demonstrated a significant advantage in reducing the risk of cervical hematoma compared to CT technique (RR 0.48, 95% CI 0.21–1.07) (Fig. 4).

#### Surgical wound infections

**Figure 5:**
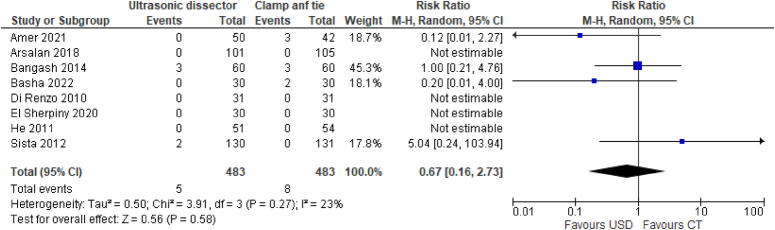
Surgical wound infections of neck.

Surgical wound infections are evaluated in 8 studies with a total of 966 participants (483 UAS and 483 CT). In the UAS technique, the risk of surgical wound infection was 1% (5/483) versus 1.6% (8/483) in the CT technique. These data show that there is no substantial difference between the two techniques (RR 0.67, 95% CI 0.16–2.73) (Fig. 5).

#### Neck seroma

**Figure 6:**
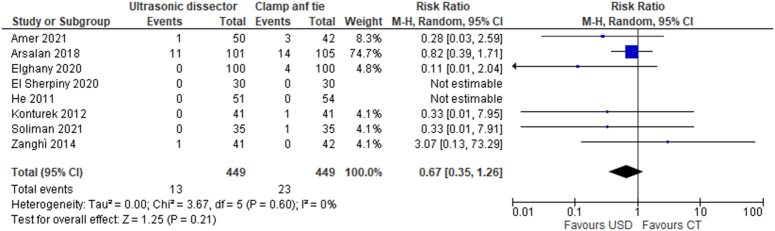
Seroma of neck.

The presence of seroma has been reported in 8 studies involving 898 patients (449 UAS and 449 CT); the risk of seroma was 2.9% in the UAS technique (13/449) versus 5.1% (23/449) in the CT technique. From the data collected, it is not possible to say that there is a significant advantage in the UAS technique compared to the CT technique (RR 0.67, 95% CI 0.35–1.26) (Fig. 6).

#### Operative time

The mean duration of surgery was assessed in 34 RCTs, with 6481 patients (3260 UAS and 3221 CT). The UAS Focus intervention has a significantly shorter mean duration than the CT technique, with a mean reduction of 27.62 minutes (*P*=0.00001). (MD=−27.62 min; 95% CI −31.84 to −23.40 min).

#### Postoperative hospitalization

**Figure 7:**
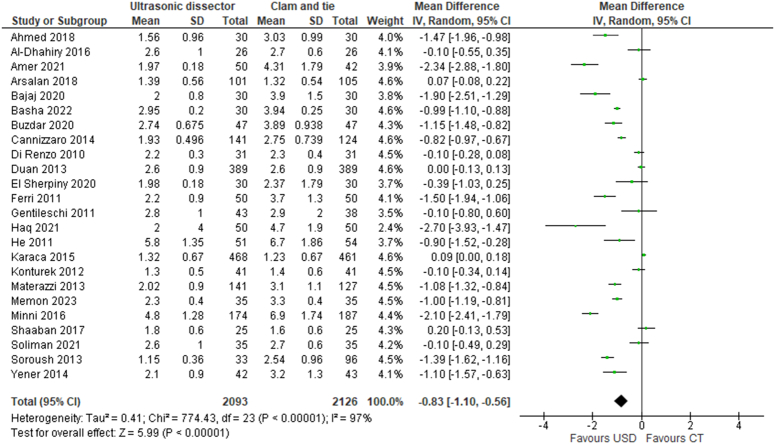
Postoperative hospitalization.

The mean length of post-surgery stay was evaluated in 24 RCTs, which considered 4219 patients; of these, 2093 patients were operated with UAS technique and 2126 with CT technique. The use of the UAS Focus technique lead to a significant reduction in post-intervention length of hospital stay compared to the CT technique, with a mean reduction of 0.83 days (MD=−0.83 d; 95% CI −1.10 to −0.56) (Fig. 7).

## Discussion

This systematic review and meta-analysis represent the largest comparison in the literature between the second-generation advanced harmonic scalpel ultrasonic focus (UAS FOCUS) thyroidectomy procedure and the conventional CT technique: 43 RCTs and 10.361 patients were included in this analysis.

The thyroid gland is a highly vascularized organ^68^, and anatomical arterial variations are fairly common^69,70^. Therefore, postoperative bleeding is one of the most important problems^71,72^. This complication is associated with cervical hematoma^73^ and the need for re-operation (0.7–1.3%)^74,75^. The most common postoperative complications associated with thyroidectomy are due to the close anatomical proximity of the thyroid gland with the recurrent laryngeal nerves and with the parathyroid glands^76^: in fact, temporary or permanent vocal cord palsy and temporary or permanent hypo-parathyroidism are frequent.

Nerve damage can occur after any intraoperative nerve injury and the outcome is often related to the severity of the injury. Minor injuries may cause transient laryngeal nerve damage, while more severe injuries can cause major nerve damage (permanent superior o inferior laryngeal nerve damage).

The incidence of permanent recurrent laryngeal nerve palsy was very low; permanent recurrent laryngeal nerve palsy was observed in 2.3% and transient palsy in 9.8%^77^.

Furthermore, the primary cause of hypocalcemia after total thyroidectomy is consequent to hypo-parathyroidism caused either by intraoperative damage to one or more parathyroid glands or their devascularisation. A systematic review of 115 studies reported that hypocalcemia is the most common postoperative complication of thyroidectomy (permanent hypocalcemia 1% and transient hypocalcemia 27%)^75^.

The greatest downside of EBDs is the high cost of these devices^78^. For this reason, decision-makers considering the purchase of a high-cost EBD should consider clinical, efficiency, and economic outcomes^79^, including the management of healthcare resources (direct and indirect costs such as operating staff and nursing time, length of hospital stay, treatment of surgical complications, re-operations, and so on)^8^.

In this review and meta-analysis, a peculiar result was identified that differs from previous published manuscripts. In fact, the incidence of transient recurrent laryngeal nerve palsy was statistically lower in patients undergoing UAS (3.99%) than in CT (5.23%) (RR 0.79, 95% CI 0.63–0.99). Cheng *et al.* ^80^ (23 studies, *n*=2204 patients) had observed that the use of Harmonic Focus was associated with fewer events of transient laryngeal nerve palsy compared to conventional thyroidectomy; however, their results were not statistically significant (RR=0.64; 95% CI: 0.28–1.44; *P*=0.28). Aires *et al.* ^81^ (24 studies with a total of 5002 patients) also concluded that there were no statistically relevant differences between the two methods with regard to transient recurrent nerve palsy, with an incidence of 4.1% in the Harmonic Focus group and 3.9% in the conventional group (95% CI from −0.01 to 0.01, *P*=0.61 and I2=0%). Revelli *et al.* ^82^ (6 studies, *n*=1374 patients) also found no significant difference between the two techniques (OR 0.42; 95% CI 0.13–1.38). The difference of results can be attributed to the sample size in our study, which avoided statistical errors related to sample under sizing (beta error 2).

The incidence of transient hypocalcemia (36 RCTs - 7041 patients) was 11.3% in patients who underwent thyroidectomy with UAS Focus and 15.4% in patients who underwent thyroidectomy with CT. We demonstrated a statistically significant benefit for patients undergoing thyroidectomy with UAS Focus compared to conventional CT (RR 0.67, 95% CI 0.56–0.81). These results confirm the conclusions already reported in literature. Cheng and colleagues (12 studies, *n*=2335 patients) demonstrated that Harmonic Focus results in a statistically significant reduction in transient hypocalcemia compared to conventional techniques in total thyroidectomy, with a RR of 0.60 (95% CI: 0.44–0.82; *P*=0.001). However, in two reviews and meta-analyses in literature, although there were slightly fewer cases of transient hypocalcemia in the UAS-treated group than in the CT-treated group, the results were not statistically significant: Aires and colleagues (25 studies, *n*=5158 patients) reported an incidence of 14.9% in patients treated with the harmonic scalpel and 15.6% in patients treated with the conventional technique (95% CI −0.04 to 0.02, *P*=0.40 and I2=56%), while in the meta-analysis by Revelli and colleagues (13 studies, *n*=1,399 patients) there were 142 cases of transient hypocalcemia in patients who underwent thyroidectomy with UAS and 137 in patients who underwent thyroidectomy with CT (OR 0.76; 95% CI 0.46–1.27).

Permanent recurrent nerve palsy and permanent hypocalcemia are the most feared complications in thyroid surgery, but fortunately their incidence is very low. The results of our meta-analysis are consistent with previous work: the incidence of permanent nerve paralysis is almost the same in both groups, while permanent hypocalcemia is slightly lower in patients who underwent UAS total thyroidectomy. The incidence of permanent laryngeal nerve palsy was evaluated in 31 RCTs, involving 6524 patients, and was 0.57% in patients who underwent thyroidectomy with UAS compared to 0.67% in patients who underwent thyroidectomy with CT (RR 1.11, 95% CI 0.51–2.42). While the incidence of permanent hypocalcemia was evaluated in 32 RCTs, involving 6689 patients, it was 0.92% in patients who underwent thyroidectomy with UAS Focus compared to 1.26% in patients who underwent thyroidectomy with CT (RR 0.80, 95% CI 0.50–1.28). In their meta-analysis, Aires and colleagues found no significant differences between the two groups, both in the incidence of permanent hypocalcemia (15 studies, *n*=3295 patients) (1.2% vs. 1.0%, 95% CI −0.01 to 0.01, *P*=0.70 and I2=0%), and in the incidence of permanent RLN paralysis (19 studies, *n*=3696 patients). (0.6% vs. 0.4%, 95% CI −0.00 to 0.01, *P*=0.50 and I^2^=0%). Cheng *et al.* also did not obtain statistically significant results for the reduction of the incidence of both permanent hypocalcemia and permanent RLN paralysis between the two methods (RR=0.35; 95% CI: 0.07–1.91; *P*=0.23; 12 studies; I^2^=0%). (RR=0.33; 95% CI: 0.01–8.03; *P*=0.50).

This systematic review and meta-analysis is the first to analyze transient and permanent superior laryngeal nerve palsy; however, despite a slight advantage of the UAS technique for prevention of transient paralysis and the CT technique for permanent paralysis, the results were not shown to be statistically significant (RR 0.95, 95% CI 0.32–2.77) and cases of permanent paralysis were analyzed in only 3 RCTs with only 672 patients (RR 1.56, 95% CI 0.27–9.14).

The use of the UAS Focus technique lead to a significant reduction in post-intervention length of hospital stay compared to the CT technique, with a mean reduction of 0.83 days (MD=−0.83 d; 95% CI −1.10 to −0.56). The reduction in complications (transient hypocalcemia, transient inferior laryngeal nerve palsy, and seromas and neck hematomas) achieved with the use of the Focus device compared to CT thyroidectomy allows for earlier patient discharge, with no additional risk.

The most statistically relevant results emerged when indirect cost indicators were compared: the mean duration of thyroidectomy was significantly shorter, with a mean reduction of 27.62 min in patients treated with the second-generation UAS Focus technique (MD=−59.52 min.; 95% CI −88.83 to −30.21 min) compared with the conventional CT technique.

Transient complications, such as temporary recurrent laryngeal nerve palsy or transient hypocalcemia, are particularly stressful for the surgeon because, despite being inherently reversible, they create significant uncertainty regarding the patient’s final outcome. This uncertainty, stemming from the fact that it is not possible to distinguish between permanent and transient complications until several months have passed, keeps anxiety levels high for both the surgeon and the patient. The clinical course remains undefined, requiring continuous monitoring and treatments that may not always lead to the desired outcome. The surgeon must manage both the patient’s anxiety and their own concern about recovery. The use of UAS Focus, by reducing the rate of transient complications such as hypocalcemia and recurrent nerve injury, indirectly decreases stress and anxiety for both the surgeon and the patient.

This study is notable for its comprehensive analysis of both transient and permanent complications associated with thyroidectomy, including the less frequently examined superior laryngeal nerve palsy. Key strengths of this research include the statistically significant reductions in transient inferior laryngeal nerve palsy and hypocalcemia linked to the use of UAS, which contrasts with previous studies that did not report such significant differences. When making surgical decisions, it is essential to consider factors such as complication rates, operative time, and hospital costs to choose the most suitable technique for each patient. The analysis reveals significant reductions in transient inferior laryngeal nerve palsy and transient hypocalcemia, along with shorter operative times and reduced postoperative hospital stays when using UAS; this advantages are indirectly associated with a cost reduction when UAS Focus is used. These findings underscore the innovative contribution of this meta-analysis to the field of thyroid surgery and highlight the advantages of incorporating UAS into clinical practice. This approach offers a strategic means of enhancing patient care and optimizing resource use.

## Conclusion

In conclusion, this study represents a comprehensive systematic review and meta-analysis of the current literature regarding thyroidectomy with the second-generation UAS FOCUS compared to the conventional CT technique.

Sutureless EBD allows for a lower risk of damage to the laryngeal nerves and parathyroid glands^62^. Moreover, the rate of hypocalcemia and nerve paresis (superior and inferior recurrent laryngeal nerves) is lower due to less thermal damage. Sutureless EBD may also reduce the rate of postoperative bleeding and cervical hematoma, a rare but potentially fatal complication. The shorter intraoperative time significantly decreases hospital costs^83^ associated with operating room utilization. However, it is important to emphasize that the differences found, although statistically significant, may not be relevant in all different economic settings.

The present study represents a comprehensive systematic review and meta-analysis of the RCTs. UAS was associated with decreased rates of transient inferior laryngeal nerve palsy, transient hypocalcemia, cervical hematoma and seroma, shorter operative time and lower postoperative length of stay. These advantages are reflected in a lower rate of transient disability, and lower hospital costs. Last but not least, they indirectly reduce the stress on the operating surgeon and the patient.

## Ethical approval

Not applicable.

## Consent

Not applicable.

## Source of funding

This research received no external funding.

## Author contribution

Conceptualization and design: R.C., V.D.A. and S.S. Data curation and analysis: R.C., M.M., E.L. and J.R. Supervision: R.C. and SS. Writing – original draft: R.C., A.A., G.P., E.L. and M.M. Writing—review and editing: R.C., E.L., I.T., D.P., S.A., G.D.T. and S.S. All Authors reviewed and approved the final version of the manuscript and fulfill the COPE (Committee on Publication Ethics) requirements for authorship.

## Conflicts of interest disclosure

The authors declare no conflicts of interest.

## Research registration unique identifying number (UIN)

Our study is a meta-analysis and a systematic review of literature: so the research registration unique identifying number is not necessary.

## Guarantor

Roberto Cirocchi.

## Data availability statement

The data used to support the finding of this study are included within the article. The data presented in this study are available on request.

## Provenance and peer review

Not commissioned, externally peer-reviewed.
